# Medical and Surgical Approaches for a Non-functioning Pituitary Adenoma During Pregnancy

**DOI:** 10.7759/cureus.55512

**Published:** 2024-03-04

**Authors:** Ach Taieb, Nassim Ben Haj Slama, Emna Mraihi, Héla Nouri, Imen Bannour

**Affiliations:** 1 Endocrinology, University Hospital of Farhat Hached, Sousse, TUN; 2 Ophthalmology, University Hospital of Farhat Hached, Sousse, TUN; 3 Gynecology, University Hospital of Farhat Hached, Sousse, TUN

**Keywords:** adenomas transsphenoidal surgery, pituitary adenomas, cabergoline, non-functioning pituitary adenomas, pregnancy

## Abstract

Non-functioning pituitary adenomas (NFPA) are most commonly found in post-menopausal women and men above the age of 50. They are mainly revealed by a tumor syndrome. The incidence of symptomatic NFPA during pregnancy is rare, with only nine documented cases in the literature.

The patient was 39 years old with no previous medical or surgical history and was 17 weeks pregnant. A large pituitary macroadenoma measuring 17 x 18 x 19 mm was discovered radiologically in the presence of a pituitary tumor syndrome. Clinical examination revealed no signs of hormone deficiency or hypersecretion. A corticotropic and thyrotropic deficit was ruled out following a hormonal workup. Ophthalmological examination revealed reduced visual acuity and bilateral visual field damage. Treatment with cabergoline at a dose of 3 mg/week was initiated following written consent from the patient. The patient underwent vaginal delivery of a healthy newborn at term. Hormonal assessment at three months postpartum definitively ruled out hormonal hypersecretion. She underwent transsphenoidal surgery, with a histological examination of the resection specimen revealing a pituitary adenoma binding adrenocorticotrophic hormone (ACTH), prolactin (PRL), and growth hormone (GH). The postoperative evaluation revealed a corticotropic and somatotropic deficit with the presence of an adenomatous residue on imaging. Substitutive treatment was then initiated along with therapeutic education.

To the best of our knowledge, this is the third case in which cabergoline treatment was initiated. Cabergoline treatment enabled the pregnancy to continue, improved the patient's clinical condition, stabilized the size of the adenoma, and prevented potential apoplexy.

## Introduction

Pituitary adenomas (PA) are a diverse group of benign tumors. Their prevalence is significantly underestimated and is reported to represent 16.7% of the general population [1]. They are categorized based on their size and functional characteristics. Non-functional pituitary adenomas (NFPA) account for 30% of pituitary masses [2]. They are generally revealed at the macroadenoma stage either by a tumor syndrome or by signs of hypopituitarism due to their non-functional nature. NFPA is uncommon in young women of reproductive age [3].

During pregnancy, the pituitary gland undergoes anatomical and physiological changes. In particular, it increases in volume by more than 120% [4]. Because of their impact on fertility, pregnancy is rarely encountered in cases with hypopituitarism. Prolactinomas are the most frequently observed PA in this context [4]. Less than 10 cases has reported the discovery of NFPA during pregnancy [3].

Managing NFPA during pregnancy can be complex and requires multidisciplinary care. The goal is to determine the type of NFPA based on the hormonal profile during pregnancy, decide on the best course of medical or surgical treatment, and plan the delivery accordingly.

Based on available literature and recommendations, this case report aims to discuss our approach to managing a patient diagnosed with NFPA during the early stages of pregnancy.

## Case presentation

A 39-year-old woman was referred to our department by the ophthalmology unit after 17 weeks of pregnancy for the management of an extensive pituitary macroadenoma revealed by tumor syndrome.

She had no previous medical history. She had had two previous pregnancies, which were incident-free and delivered vaginally. Her menstrual cycles were regular. However, she reported the onset of galactorrhea and cycle dysfunction three months before pregnancy.

The development of symptoms occurred at 16 weeks of pregnancy following a stressful event. Symptoms included blurred vision and decreased visual acuity associated with headaches. Ophthalmological examination revealed a dynamic visual acuity (DVA) of 3/10, visual field impairment, and bilateral stage I papilledema.

**Figure 1:**
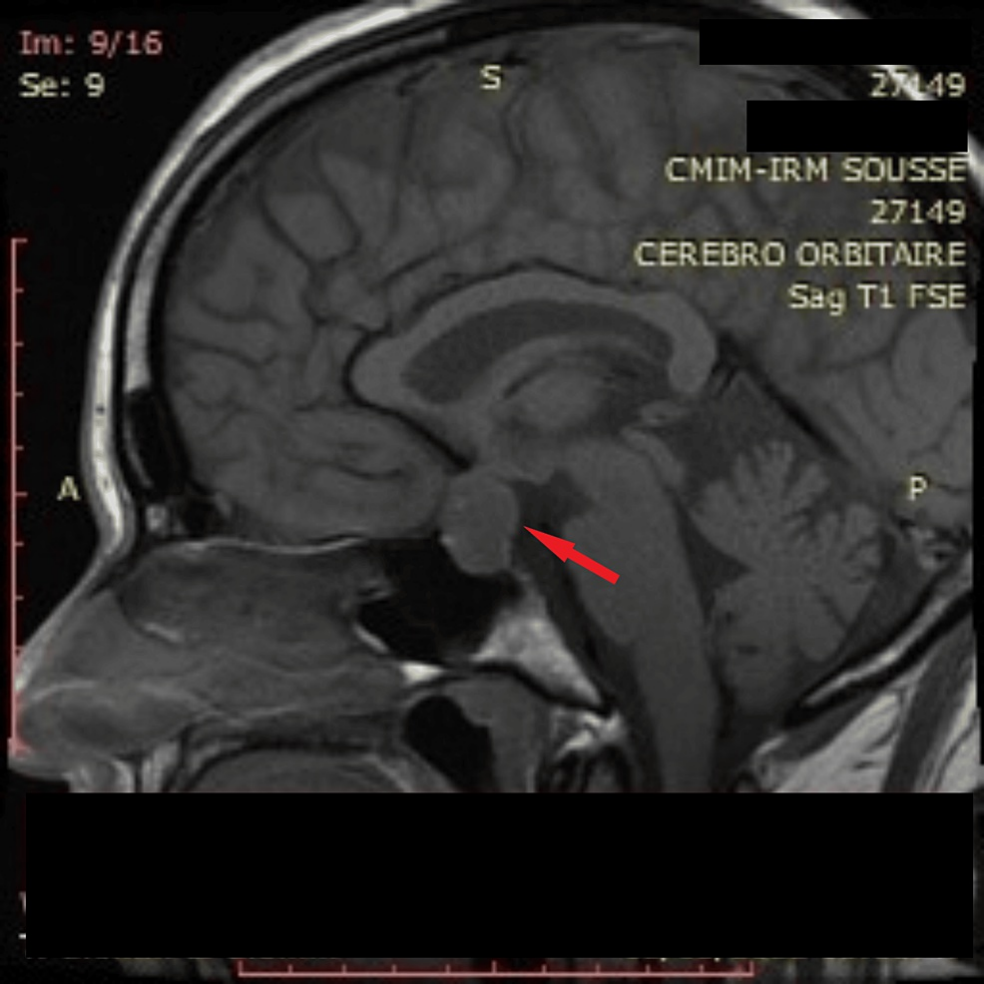
Preoperative hypothalamic-pituitary MRI sagittal section T1-weighted sagittal section of a 17 x 18 x 19 mm pituitary adenomatous lesion (red arrow).

**Figure 2:**
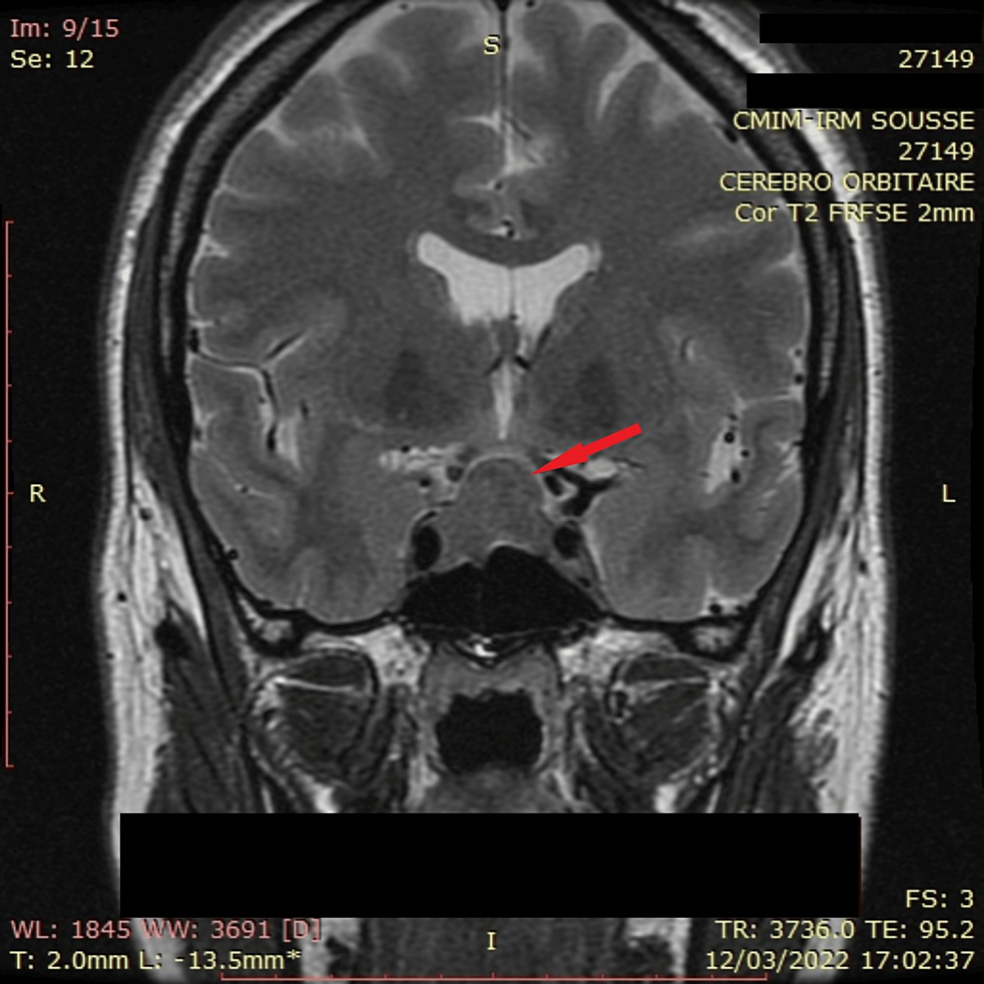
Preoperative hypothalamic-pituitary MRI coronal section T2-weighted coronal section of pituitary adenoma showing suprasellar extension with compression of the optic chiasm and inferior extension in contact with the right cavernous sinus, which includes the carotid artery (Knosp 2) as indicated by the red arrow.

A gadolinium-free MRI was then performed urgently, revealing a 17 x 18 x 19 mm PA with an enlarged pituitary gland. There was a suprasellar extension with mass effects on the optic chiasm and inferior extension at the roof level of the right sphenoidal sinus in contact with the right cavernous sinus without compression of the latter and encompassing the carotid artery (Grade Knosp 2) (Figures 1, 2).

There was no physical evidence of acromegaly or Cushing's disease. Pituitary apoplexy was ruled out, and an evaluation of anterior pituitary function was conducted to search for potential deficits. The results of the blood workup were as follows: thyroid-stimulating hormone (TSH): 1.1 mUi/L (0.5-4.5 mUi/L), thyroxine (FT4): 14.3 pmol/L (9-19 pmol/L), and basal cortisol: 320 ng/mL (100-380 ng/mL). Her thyroid and corticotropic functions were normal. Prolactin (PRL) was 225 ng/mL. Surgery was considered, but the patient did not accept transsphenoidal surgery.

Treatment with cabergoline was progressively initiated up to a dose of 3 mg/week. Given the extensive nature of the disease and the risk of apoplexy, treatment with hydrocortisone at a dose of 15 mg/day was also initiated.

During follow-up, the patient's tumor syndrome regressed, and her visual field normalized, with improvement in visual acuity to 7/10 bilaterally and the disappearance of papilledema. On the MRI scan, there was no change in the size of the pituitary macroadenoma. The patient had an incident-free vaginal delivery in August 2022, at 39 weeks of pregnancy (WP), delivering a healthy male newborn. The continuation of cabergoline treatment inhibited the onset of lactation. Three months postpartum, hormonal assessment ruled out Cushing's disease and acromegaly. PRL was 7 ng/ml (measured under cabergoline).

Six months after delivery, transsphenoidal excision of the PA was performed. Histological and immunohistochemical examination of the excised specimen revealed that PA expressed adrenocorticotropic hormone (ACTH) at 50%, PRL at 20%, and rare growth hormone (GH) cells. Regarding proliferation factors, Ki67 was 2%, P53 was negative, and mitoses were absent.

During the three-month postoperative assessment, it was discovered that the tumor syndrome had disappeared, and visual acuity and visual field had returned to normal. An evaluation of the anterior pituitary functions was carried out, which included assessing the corticotropic and somatotropic axes using the insulin tolerance test (Table 1).

**Table 1:** Assessment of the anterior pituitary functions in the patient three months post transsphenoidal surgery ACTH: Adrenocorticotropic hormone; FSH: Follicle-stimulating hormone; GH: Growth hormone; FT4: Thyroxine; ITT: Insulin tolerance test; LH: Luteinizing hormone; PRL: Prolactin; TSH: Thyroid-stimulating hormone.

	Result	Reference range
Peak of cortisol during ITT (ng/mL)	131	>180
ACTH (pg/mL)	5.9	<50
Peak of GH during ITT (ng/mL)	1.94	>5
PRL (ng/mL)	10	5-20
TSH (mUI/ml)	1.21	0.25-5
FT4 (pmol/l)	14.15	9-22
Estrogen (ng/ml)	50	30-200
FSH (UI/L)	4.57	4-12
LH (UI/L)	3.81	2-10

**Figure 3:**
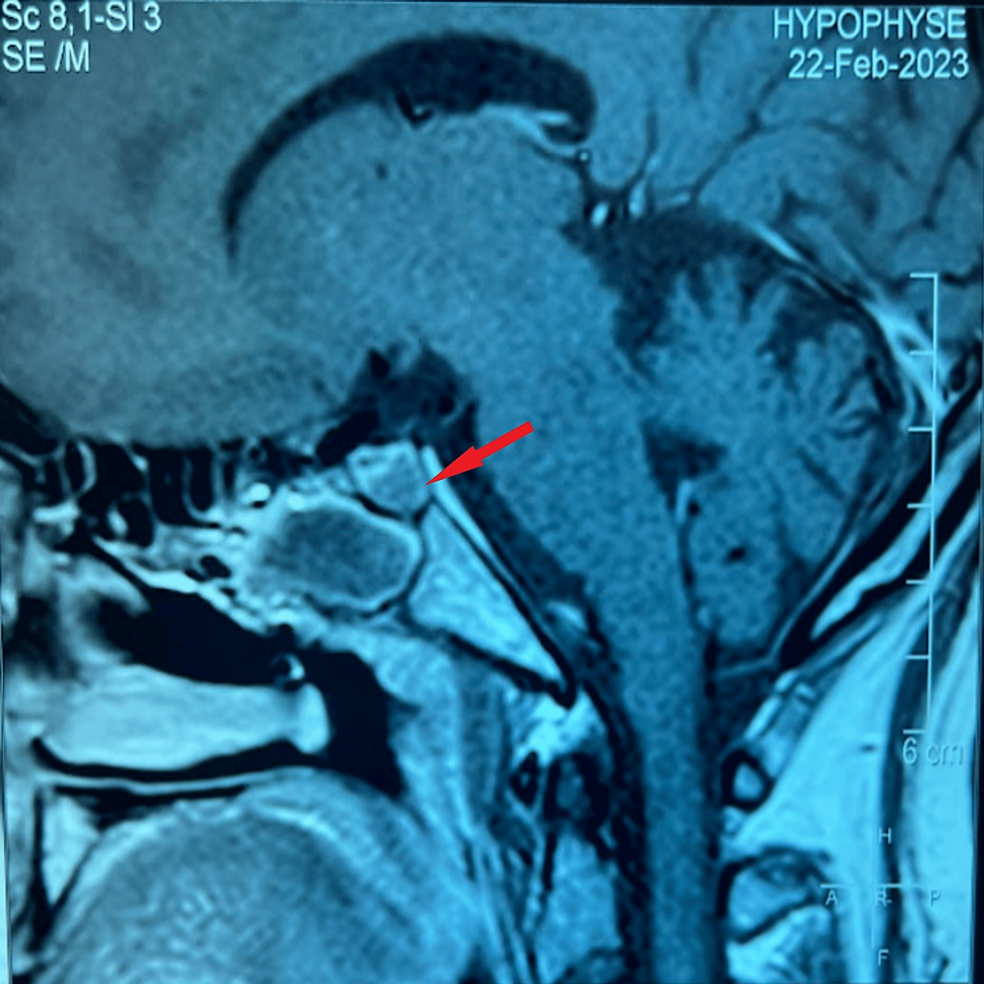
Postoperative hypothalamic-pituitary MRI sagittal section T2-weighted sagittal section of an 8 x 6 x 5 mm pituitary adenomatous residue with postoperative status and fat-filling material (red arrow).

**Figure 4:**
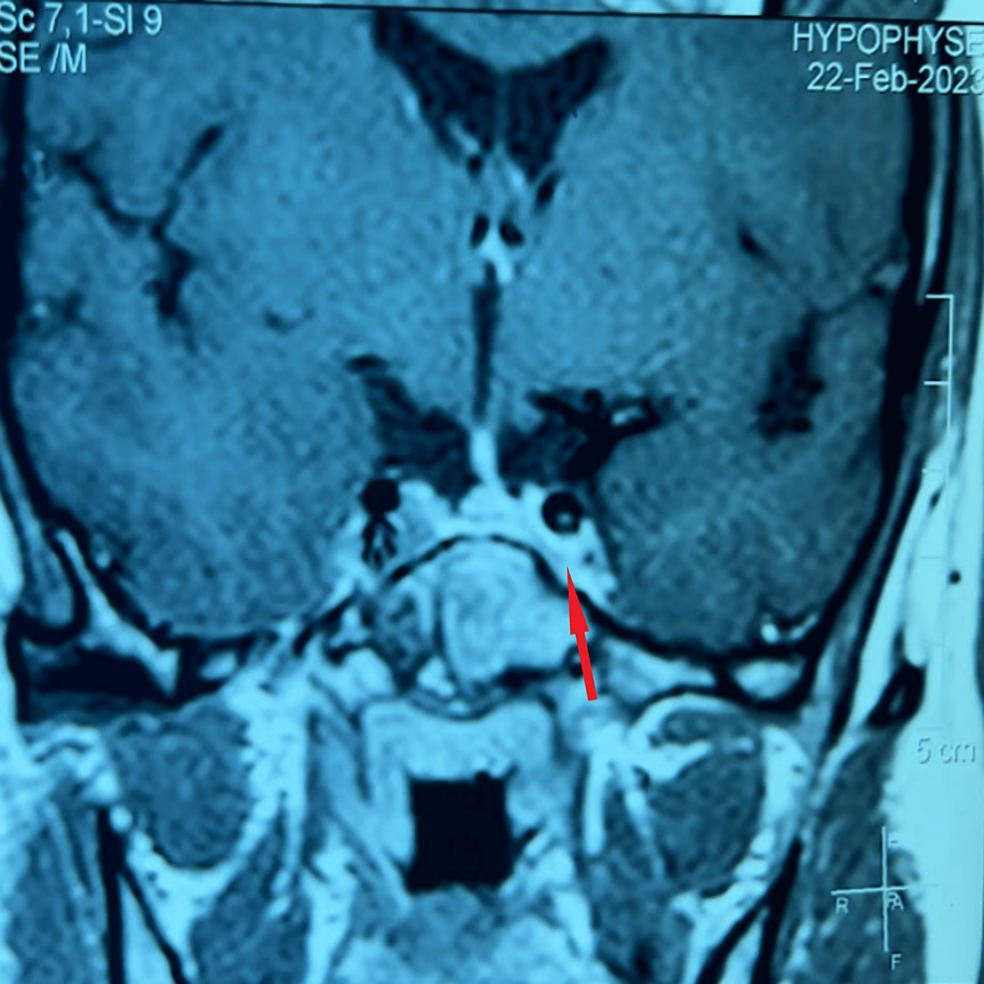
Postoperative hypothalamic-pituitary MRI coronal section T2-weighted coronal section of the pituitary adenomatous residue which comes into contact with the intracavernous portion of the right carotid sinus artery (red arrow).

A hypothalamic-pituitary MRI revealed that an 8 x 5 mm residue was still present and in contact with the cavernous portion of the right carotid artery (Figures 3, 4). The patient was prescribed a substitutive treatment of hydrocortisone at a dose of 15 mg/day, which was maintained with a clinical-biological check-up after six months.

## Discussion

NFPA has a prevalence of 50 to 60 cases per million inhabitants per year and an annual incidence of four to five cases per million inhabitants [5]. They are most commonly found in post-menopausal women and men above the age of 50 [6]. Due to their non-secretory nature, they are usually discovered by chance during brain imaging or as part of tumor syndrome [7]. The discovery of PA in a pregnant woman remains a rare occurrence. Fertility is frequently impaired by PA due to hormonal hypersecretion or mass effect resulting in hypopituitarism [4]. In a review of the literature, only 31 pregnancies were associated with NFPA, with only nine symptomatic NFPAs revealed during gestation. Six of the nine symptomatic NFPA were revealed by tumor syndrome [3].

During pregnancy, the pituitary gland increases in size from the first WP due to hyperplasia of lactotrophic cells resulting from increased placental secretion of estrogen [4]. This increase in size in our patient was responsible for the tumor syndrome, the ophthalmological involvement, and the extensive nature of the NFPA on imaging.

The main emergency in this case was to rule out pituitary apoplexy using hypothalamic-pituitary imaging. Although rare, this complication can be fatal in pregnant patients. In a review of the literature on this subject, 48 cases of pituitary apoplexy occurred in pregnant women, with one death reported [8]. After prolactinomas, NFPAs were the second most common cause of pituitary apoplexy during pregnancy, accounting for 35% of cases.

Apoplexy having been ruled out in this case, the second step is to detect any anterior pituitary deficiencies. For the thyroid axis, a TSH and FT4 assay was therefore necessary. For the corticotropic axis, the peak cortisol values during the synacthen test are higher because of the physiological hypercorticism during pregnancy. A cortisol peak of >290 ng/ml during the ST test in the second trimester is necessary to rule out adrenal insufficiency [9].

The next step was to decide whether the PA was secretory. However, due to the physiological variation in hormonal secretion during gestation, it is generally difficult to decide whether the PA is functional. For this reason, it is generally recommended to perform these tests three months postpartum [4]. The final step was to decide on the course of treatment. The latest recommendations favor surgery if tumor reduction of PA is required during pregnancy.

However, treatment with cabergoline could also be considered [4]. Since the patient rejected the surgical decision, we decided to gradually initiate treatment with cabergoline up to a dose of 3 mg/week. A review of the literature was carried out by Lin et al. on the treatment of NFPA with cabergoline. They concluded that a cabergoline dose of 1-3 mg/week for six to 12 months stabilized the size of NFPA in more than 80% of cases [10]. There is less data on the safety of cabergoline during pregnancy than on bromocriptine [4]. Nevertheless, various studies have concluded that the teratogenic profile is similar and cabergoline is preferred to bromocriptine for its superior antitumor effect [4]. Treatment with hydrocortisone was nevertheless initiated given the high risk of apoplexy, particularly after the introduction of cabergoline, which is considered to be a risk factor for apoplexy [11]. Following the recommendations and after multidisciplinary consultation with the intensive care unit and the gynecologists, the decision was made to deliver the patient vaginally without an epidural at 39 WP [4]. This decision was based in particular on the lower risk of apoplexy during vaginal delivery compared to cesarean section [8].

After postpartum resection of the NFPA, pathological examination of the surgical specimen revealed PA expressing ACTH (50%), PRL (20%), and rare GH cells. However, as research into transcription factors is not available in our country, it is not possible to classify PA according to the latest recommendations of the World Health Organization [12]. However, it is likely to be a plurihormonal PA, given the immunohistochemical profile. This case has a poor prognosis with a high risk of recurrence due to the invasive nature and initial size, the young age, the presence of an adenomatous residue, and the probable plurihormonal nature of the PA [13]. Long-term follow-up is therefore necessary.

## Conclusions

According to our knowledge, this is the 10th case of symptomatic NFPA discovered during pregnancy and the third case of NFPA treated with cabergoline during pregnancy. In our patient, cabergoline treatment improved the tumor symptoms, stabilized the size of the pituitary adenoma, postponed surgery until after delivery, and prevented potential apoplexy. A larger number of case reports similar to ours will be required to establish reliable recommendations based on a high level of evidence. This will help in better management of patients and improve their prognosis.
